# HBV in Italian Women’s Jail: An Underestimated Problem?

**DOI:** 10.3390/jcm13051398

**Published:** 2024-02-28

**Authors:** Nicholas Geremia, Federico Giovagnorio, Andrea De Vito, Luca Martignago, Vito Fiore, Elena Rastrelli, Giordano Madeddu, Saverio Giuseppe Parisi, Giulio Starnini, Sandro Panese, Sergio Babudieri

**Affiliations:** 1 Unit of Infectious Diseases, Department of Clinical Medicine, Ospedale Dell’Angelo, 30174 Venice, Italy; sandro.panese@aulss3.veneto.it; 2 Unit of Infectious Diseases, Department of Clinical Medicine, Ospedale Civile “S.S. Giovanni e Paolo”, 30122 Venice, Italy; 3 Department of Molecular Medicine, University of Padua, 35121 Padua, Italy; federico.giovagnorio@studenti.unipd.it (F.G.); luca.martignago@aulss3.veneto.it (L.M.); saverio.parisi@unipd.it (S.G.P.); 4 Unit of Infectious Diseases, Department of Medicine, Surgery and Pharmacy, University of Sassari, 07100 Sassari, Italy; andreadevitoaho@gmail.com (A.D.V.); vitofiore30010516@gmail.com (V.F.); giordano@uniss.it (G.M.); babuder@uniss.it (S.B.); 5 School in Biomedical Science, Biomedical Science Department, University of Sassari, 07100 Sassari, Italy; 6 Medicina Protetta-Unit of Infectious Diseases, Belcolle Hospital, 01100 Viterbo, Italy; elena.rastrelli@gmail.com (E.R.); hepan007@gmail.com (G.S.)

**Keywords:** women prisoners, HBV, viral hepatitis

## Abstract

Background: There is little information regarding the hepatitis B virus (HBV), vaccination status, and hepatitis B exposure in Italian women’s jails. We aimed to describe the HBV exposure and HBs antibody (anti-HBs) protection levels in female prisoners. Material and methods: A retrospective multicentric study was performed in Italian prisons from 2021 to 2023. Univariate and multivariate analyses were conducted to identify risk factors for HBc antibody (anti-HBc) seropositivity and non-protective anti-HBs titer. Results: We included 156 patients. The median age was 41.0 (IQR 34.0–48.0). Of the studied subjects, 31 (19.9%) had anti-HBc positive titer. Two women were HBsAg positive. In the multivariate analysis, older age [OR 1.06 (CI 1.01–1.11), *p* = 0.011], North-Eastern European [OR 11.67 (3.29–41.30), *p* < 0.001] and African origin [OR 6.92 (CI 1.51–31.60), *p* = 0.013], and drug use [OR 6.55 (CI 1.96–21.9), *p* = 0.002] were risk factors for HBV exposure. Thirty-seven (32%) women had no history of HBV vaccination. Forty-four (38%) had an anti-HBs non-protective titer. In the multivariate analysis, North-Eastern European origin [OR 4.55 (CI 1.19–17.50), *p* = 0.027] was associated with unprotective anti-HBs titer. Conclusion: Our results show both the low prevalence of HBV and protection in female prisoners. Age, North-Eastern European and African origin, and drug use have a role in exposure risk to HBV.

## 1. Introduction

Hepatitis B virus (HBV), a DNA virus belonging to the family Hepadnaviridae, represents a significant global public health challenge. Chronic HBV infection, which currently affects approximately 297 million individuals worldwide, including over 6 million children under the age of 5, can lead to hepatocarcinoma [1,2]. The main ways of HBV transmission include injective drug use and unprotected sexual contact. Still, transmission can also occur in many developing countries through unsterilized therapeutic procedures [3,4] and, significantly, mother-to-child transmission (MTCT). MTCT remains a substantial challenge in these regions, with a high transmission rate without appropriate prophylactic interventions [5].

The cornerstone of HBV eradication is preventive vaccine coverage, followed by adequate diagnostic and treatment programs [6,7,8,9].  Several vaccines are available for the primary prevention of HBV, providing adequate serologic protection in 90 to 95 percent of cases [10]. However, studies indicated that older adults (>40 years of age) are less likely to achieve a seroprotective response to HBV vaccination and this drops to 60–70% in adults aged 60 years and older [11]. People with HBs antibody (anti-HBs) titer inferior to 10 mUI/mL are considered non-responsive to the vaccine, and insufficient anti-HBs titers may fail to prevent HBV transmission [12]. In May 2016, the World Health Organization (WHO) adopted a global hepatitis strategy to eliminate viral hepatitis by 2030 [13]. The targets to be achieved include a 90% reduction in new cases of chronic hepatitis and a 65% reduction in mortality due to infections, which rely on 80% of treatment-eligible individuals with chronic HBV and HCV infections being treated globally [14]. Some studies evaluate vaccination in high-risk settings as an appropriate and cost-effective measure to prevent HBV’s severe manifestation and death. Hall et al. demonstrated that prevaccination screening and testing with the first dose of a two-dose vaccination series at the initial encounter averted chronic HBV infections with a cost-saving profile of $266 per person in jail [15].

Current antiviral treatments of chronic hepatitis B (CHB), represented by Peg-interferon-α and Nucleos(t)ide Analogues (NA) achieve a functional cure of HBV infection, defined as HBsAg and HBV DNA clearance, in a limited number of patients [16]. For this reason, the need to fulfill the tertiary prevention and reach the HBV eradication goal with novel agents is mandatory. A new point of view in treating HBV targeting the capsid protein of the virus and interrupting the normal capsid formation has led to the conception of advanced drugs [17,18,19]. The preliminary data collected in vitro and in vivo in animal models are promising, suggesting the possibility of clinical introduction. 

Prisons and jails are potential reservoirs of viral hepatitis due to higher rates of blood-borne infection (BBI) among detainees, including HBV, HCV, and HIV [20,21]. This increased risk is attributed to the concentration of marginalized people, such as people who inject drugs (PWID) and people who exchange sex for drugs, or sex workers [21,22,23]. A high prevalence of parenterally and sexually transmitted infections was found among inmates of Italian prisons [24]. HIV and HCV were found in 7.5% and 38.0% of the inmates, respectively. HBsAg was present in 7% of the population. Evaluating anti-HBc titer, nearly 53% of the Italian detainees had this marker of past infection, underlying a high proportion of viral circulation [24].

In Italy, there are 190 correctional facilities: among these, there are 52 isolated departments and only 5 of the 190 structures are women’s prisons located in Trani, Pozzuoli, Roma Rebibbia, Empoli, and Venezia Giudecca [25]. Of the 56.127 prisoners in Italy, 2392 are women, representing 4% of the inmate population [26,27]. The total number of foreign detainees is 17.687, of which 722 (4%) are women [26,27].

Incarcerated women, often more vulnerable due to drug use, socio-economic challenges, mental health issues, and a higher incidence of abuse compared to men, represent a particularly high-risk group [28]. For these reasons, vaccination of high-risk female adults, especially those who are incarcerated, is crucial and often represents their only opportunity for HBV screening and follow-up [29,30]. It also represents a unique opportunity to prevent the risk of HBV circulation in micro-reservoirs, as in the prison case [31,32]. Despite the importance of understanding HBV vaccination coverage in prison populations, data are limited and show significant geographical variability. Current published literature indicates that people in detention are under-immunized, particularly against HBV, with profound variability worldwide [33,34].

Regarding HBV infections and the seroprevalence of anti-HBs protective titers in incarcerated women, the data suggest similarities to the male incarcerated population, with high rates of HBV susceptibility to infection individuals [35]. To our knowledge, available national HBV data about females in detention are scarce, and extrapolating information is challenging due to the relatively low proportion of women in Italian prisons (range 2.1–6.9%) [21].

The primary aims of our research were to evaluate the prevalence of HBV infection, exposure to the virus, and serological HBV protection among female detainees. The secondary aims were to study this group’s risk factors associated with HBV exposure and seroprotection.

## 2. Materials and Methods

We conducted a retrospective multicentric study in Italian correctional facilities. Data were collected from January 2021 to December 2023.

### 2.1. Inclusion Criteria and Variable Definitions

**Figure 1:**
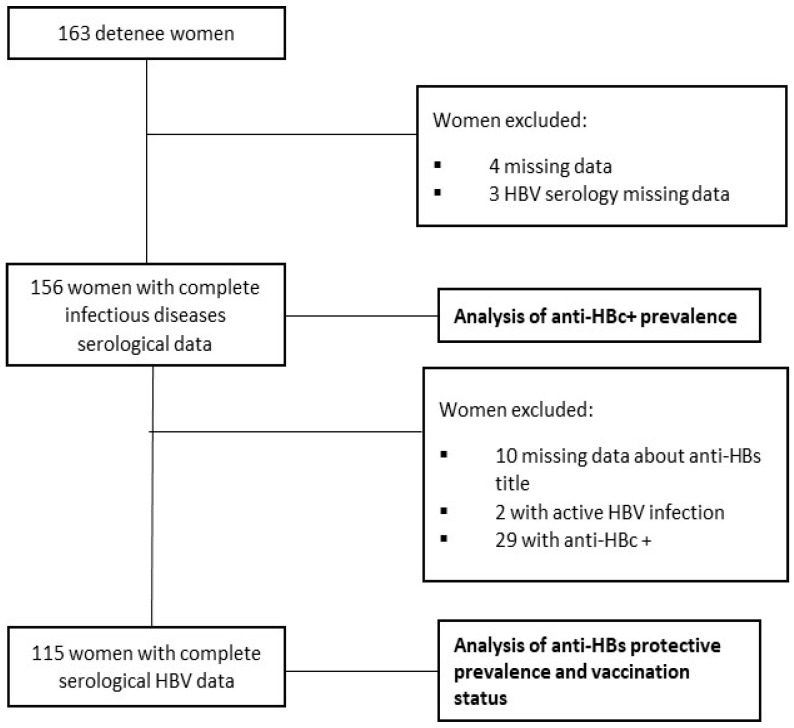
Flow chart of the study.

We enrolled adult women who were ≥18 years old and addressed in correctional settings. The exclusion criteria were age below 18 years old, women who were on probation, and detainees in maximum security isolation. The study was conducted in two divided datasets. The first analysis focused on the prevalence of anti-HBc, excluding participants with incomplete serological data for HBV or other baseline BBI diseases, such as HCV, HIV, and syphilis infection. The HBV–HDV coinfection could not be studied due to the impossibility of conducting such screening in jail. The second analysis examined the prevalence of anti-HBs titer protection, excluding women with unknown anti-HBs titer, inmates with active HBV infection, and individuals with HBc antibody (anti-HBc). The research flowchart is summarized in Figure 1.

Complete HBV serology includes HBs antigen (HBsAg), anti-HBs with titer, and anti-HBc.

HBV infection was defined by the presence of HBsAg and anti-HBc reactive titer or by HBsAg negative status and anti-HBc reactivity in women under HBV treatment. HBV exposure was defined as the presence of anti-HBc. Seroprotection was determined as an anti-HBs titer ≥10 mUI/mL without anti-HBc. The participants were considered unvaccinated if the anti-HBs titer was equal to 0 mUI/mL.

Active drug abuse status was considered if the detainee had a history of injecting drug use and documented data from the toxicological urine tests in the year before incarceration.

### 2.2. Analyzed Parameters

Data were divided into demographical (age and areas of origin), serological infectious baseline status (coinfections with HIV, HCV, and syphilis), and behavioral parameters (risk factors for contracting infections, such as drug abuse and substitutive drug therapy). Infectious blood and sexually transmitted diseases were checked at the time of incarceration. These included anti-HCV and HIV tests, HBV serology profiles, and syphilis screening. In the reactive status of the anti-HCV and HIV test, polymerase chain reaction (PCR) was performed to identify HCV RNA, genotype, and HIV RNA. If HBsAg was positive, HBV DNA was checked. Positive syphilis screening led to second-level testing with Venereal Disease Reference Laboratory (VDRL) and Treponema Pallidum Hemagglutination Assay (TPHA). Screening for HIV was conducted with the Abbott Architect™ (Chicago, IL, USA) HIV Ab/Ag Combo assay, a chemiluminescent microparticle immunoassay (CMIA); research on HCV antibodies was performed with an Abbott Architect™ anti-HCV CMIA; syphilis screening was conducted with the Abbott Architect™ i2000SR CMIA; measurement of HBsAg, anti-HBs, and anti-HBc was performed using Abbott Architect™ i2000SR CMIA. The HBV DNA was controlled with the Abbott HBV RealTime assay, an in vitro polymerase chain reaction (PCR); the HCV RNA was searched with the Abbott m2000™ RealTime assay; the HIV RNA was checked with Abbott Real-Time™ HIV1 Viral Load Assay. 

### 2.3. Statistical Analysis

Data were collected with Excel (Microsoft, Redmond, WA, USA). We evaluated the normality of the distribution for quantitative data using the Shapiro–Wilk test. Quantitative variables were described with the median and interquartile range (IQR), or mean with standard deviation (SD), according to the normality of the distribution. Qualitative variables were summarized with absolute and relative (percentage) frequencies. Continuous variables were compared with the Mann–Whitney U test or Student *T*-test, according to the normality of the distribution. Categorical variables were evaluated with Chi-squared or Fisher’s exact test, as appropriate. Univariate logistic regression assessed risk factors related to anti-HBc+ and non-protective anti-HBs status, defined as an HBs titer < 10 mUI/mL without a previous history of an HBs titer that reached and/or exceeded the 10 mUI/mL value after the complete vaccination. A stepwise approach was conducted to multivariate logistic regression to determine the same parameters. It was considered a confidence interval (CI) of 95%, and a *p*-value (*p*) ≤ 0.05 indicated statistical significance. Statistical analysis was conducted with STATA version 16 (StatsCorp, Lakeway, TX, USA).

### 2.4. Ethics Issues

All enrolled women provided informed consent for inclusion before participating in the study. The study was conducted following the Declaration of Helsinki. All studies regarding the Italian Penitentiary System have been approved by the Ethics Committee of the University of Rome “Tor Vergata” [Registro sperimentazioni 73/05].

## 3. Results

### 3.1. Anti-HBc Reactive Prevalence

**Figure 2:**
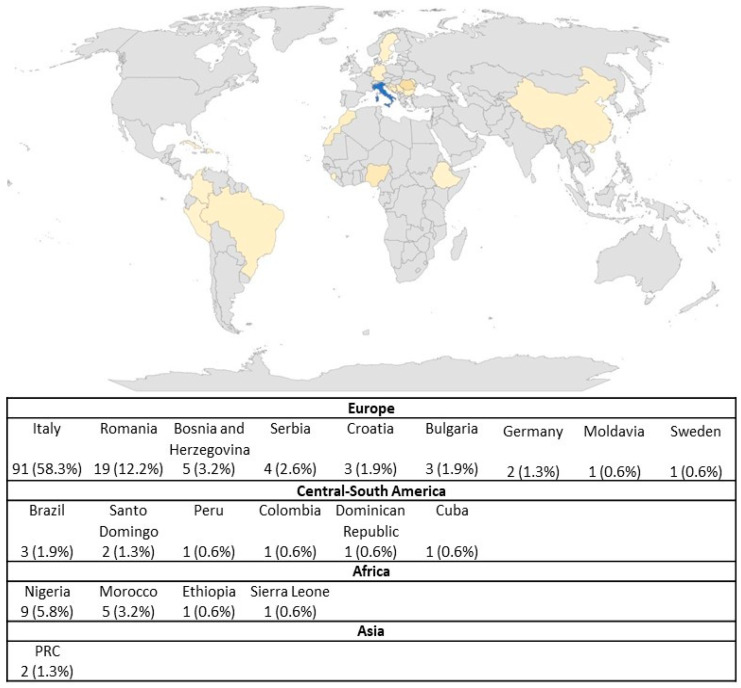
Countries of origin of the female detainee population included in the study. PRC = People’s Republic of China.

A total of 156 incarcerated females were included. Of these, 31 (19.9%) tested positive for anti-HBc, and 2/31 (6.5%) were also positive for HBsAg. The median age of the participants was 41.0 (IQR 34–48) years. The majority, 91 (58.3%), were Italian. The most represented European countries were Romania (19 participants, 12.2%), Bosnia and Herzegovina (5 participants, 3.2%), and Serbia (4 women, 2.6%). Among the African participants, the largest group was from Nigeria (9 participants, 5.8%) and Morocco (5 women, 3.2%). Details on other countries of origin are provided in Figure 2. 

Regarding the HCV status, 33 (21.1%) women tested positive for HCV antibodies. Of these, 25 (75.8%) were anti-HBc negative and 8 (24.2%) were anti-HBc positive. HCV RNA testing revealed that 16 (48.5%) had an active HCV infection; of them, 13 (81.2%) were anti-HBc negative and 3 (18.8%) were anti-HBc positive.

Concerning HIV status, only 4 (2.6%) women tested positive, all of whom were anti-HB negative. All the participants tested negative for VDRL, and only 4 (2.9%) tested positive for TPHA, indicating past exposure to syphilis. 

Unprotected sexual intercourse was identified as the most frequent risk factor for HBV transmission, according to the patients’ anamnesis. The second most common transmission risk was the use of injective drugs (28 women, 18.0%). The general characteristics of the study population are reported in Table 1.

### 3.2. Univariate and Multivariate Analysis about Risk Factors for Anti-HBc + Status (HBV Exposure)

The univariate analysis assessed five parameters: age, geographical area of origin, presence of anti-HCV positive test, way of HBV transmission, and whether there was substitutive therapy or other psychiatric treatment (active use of methadone or benzodiazepine). Older age [OR 1.03 (CI 1.00–1.07), *p =* 0.05], Eastern European origin [OR 4.24 (CI 1.97–10.60), *p* = 0.002] and people who inject drugs (PWID) [OR 2.57 (CI 1.01–6.52), *p* = 0.047] were associated with an increased risk of HBV exposure. In the multivariate analysis, older age [OR1.06 (CI 1.01–1.11), *p* = 0.011], Eastern European origin [OR 11.67 (3.29–41.30), *p* < 0.001], African origin [OR 6.92 (CI 1.51–31.60), *p* = 0.013] and being a PWID [OR 6.55 (CI 1.96–21.9), *p* = 0.002] were identified as risk factors for an anti-HBc positive status. Univariate and multivariate analysis is reported in Table 2.

### 3.3. Serological Prevalence of Anti-HBs Protective Titer

**Figure 3:**
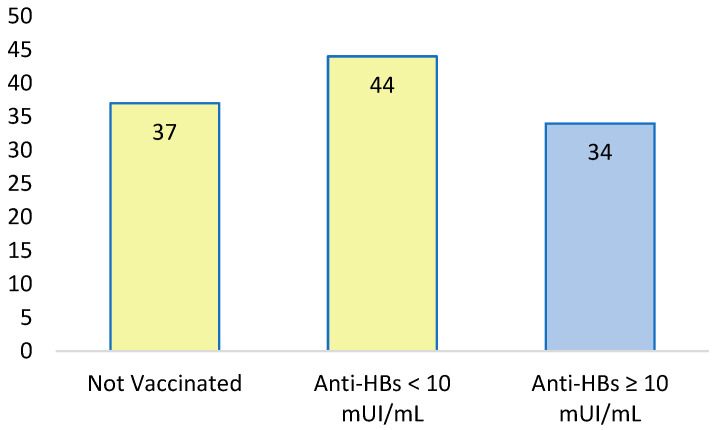
Absolute values of anti-HBs vaccination status and seroprotection. In light yellow, people with an anti-HBs titer ≤ 10 mUI/mL and no vaccination; in light blue, protective anti-HBs titer.

We included 115 female detainees with complete HBV serological data. Among them, 37 (32%) were completely unvaccinated, while 44 (38%) had a non-protective anti-HBs titer. The distribution among vaccinated, non-vaccinated, and vaccinated subjects with non-protective antibody titer is illustrated in Figure 3.

The median age of the participants was 39.0 (IQR 32.0–48.9). The majority of incarcerated women were Italian, 71 (61.7%). The non-protective anti-HBs cohort was represented by 47 Italians (40.9%), 21 North-Eastern Europeans (18.3%), and 10 Africans (8.7%). Twenty (17.4%) women had a positive HCV antibody test. HCV RNA testing was positive in 7 (35.0%) cases. The HIV test was reactive in only 2 (1.7%) participants. Also, in this cohort, sexual intercourse remains the most frequent risk factor underlying the potential transmission of HBV. Seventeen women (14.8%) had a history of using injective drugs. The general characteristics of the population are shown in Table 3.

### 3.4. Univariate and Multivariate Analysis about Risk Factors for Non-Protective Anti-HBs Titer

The univariate analysis examined factors such as age, geographical area of origin, HCV and HIV infection, risk factors for BBI, and substitutive therapy with methadone. In the multivariate analysis, only North-Eastern European origin [OR 4.55 (CI 1.19–17.50), *p* = 0.027] was associated with the risk of not vaccination or anti-HBs titer < 10 mUI/mL. Univariate and multivariate analysis is reported in Table 4.

## 4. Discussion

Much has been written about male incarcerated patients, while little about females. Gender, ethnicity, and country of origin could impact the risk of BBI, immunological response, behaviors, and social conditions. In our cohort, North-Eastern European birth was associated with HBV exposure and anti-HBs non-protective status. 

Considering in detail the country of origin of our population, most inmates were from the Balkan area. This result corresponds with the patterns of HBV vaccination diffusion in Europe. In the European Union (EU), 27 countries recommend universal childhood vaccination against HBV. Data on vaccine coverage in 2020 were available from 23 countries. Of these, only 11 countries (50%) have met the target of 95% coverage [36]. Some countries have reported declining HBV vaccination rates since 2019 due to the COVID-19 pandemic. In particular, Romania (−3%), Bulgaria (−2%), and Croatia (−2%) reported the most significant declines [37].

Additionally, there is considerable variation among countries regarding HBV infection data. While Western Europe has a low disease prevalence, Eastern Europe presents a more diversified epidemiology accounting for countries with a “high-intermediate” HBV endemicity [38,39,40]. According to Naikitanda et al., HBV screening and HBV vaccination in prisoners across Europe vary significantly from country to country, indicating a lack of comprehensive data from various nations and highlighting how eligibility varies, ranging from mandatory to only recommended [41]. It is known in the literature that the prevalence of viral hepatitis within prisons is much higher than in the general population [42]. In European jails, the HBV infection ranged from 0% in a maximum-security prison in the United Kingdom (UK) to 25.2% in two Bulgarian juvenile detention centers [41]. 

In Africa, HBV is endemic, and many countries are developing viral hepatitis management guidelines and strategic plans to contrast the diffusion of viral hepatitis. The prevention of MTCT is essential to reduce HBV transmission in Sub-Saharan Africa [14]. Since the 1990s, African countries have gradually introduced the HBV immunization. All countries have adopted a three-dose infant vaccination. In contrast, only 14 countries currently provide a monovalent birth-dose HBV vaccine [43]. The regions with the highest endemicity (more than 8% of the population) in Sub-Saharan Africa include Nigeria, Namibia, Gabon, Cameroon, and Burkina Faso [44]. Other countries, such as Egypt, Tunisia, Algeria, and Morocco, have a 2–7% prevalence of HBV. As in other parts of the world, also in African jails, inmates have an HBV prevalence higher than the general population [45], with a seroprevalence of HBsAg ranging between 12% and 17.4% [45,46,47,48]. According to these data, our African cohort was most represented by Nigerians and Moroccans, where HBV exposure is much higher than in other African countries [44,46].

Regarding the country of origin, our study found that individuals from Africa and Eastern Europe had a higher risk of HBV exposure [49]. This trend aligns with data observed in the non-incarcerated population. Otherwise, according to data from the Italian prison administration department, in 2023, foreign inmates represented 31.3% of the population. Foreign women present in Italian prisons constitute 4.1% of the total number of detained and 29.6% of the women in jail. The most represented nationalities among foreign women in Italian correctional institutes are Romanian (25.9%), Nigerian (13.6%), Bosnian (5.9%), Moroccan (4.6%), and Bulgarian (4.2%) [50].

In Italy, the vaccination campaign has been characterized by two phases. From 1984 to 1991, plasma-derived vaccines were performed in high-risk individuals. From 1991, the recombinant vaccine targeted was administrated to individuals between two months and twelve years [51]. In our study, the correlation between age and increasing seroprevalence of the anti-HBc positive status is understandable. Clearly, given the absence of vaccination against HBV before 1984, the older population was at greater risk of having been exposed to the virus at a young age. The impact of the vaccination campaign in Italy on HBV circulation has been significant, with a progressive decrease in HBV seroprevalence. Notably, the incidence of acute HBV infection dropped from 10.4 cases per 100,000 inhabitants in 1987 to 5.4 in 1990, then 0.9 in 2012, 0.6 in 2016, and 0.21 in 2020 [52]. 

In terms of HBV exposure, when compared to international studies conducted in correctional institutes and jails, the prevalence of anti-HBc positivity found in our study was higher than in Iran (7.4%) and Brazil (9.8%), and lower than in Switzerland (32.4%), Australia (21.7%) and Spain (30.4%) [35,53,54,55,56]. Furthermore, our results showed a lower prevalence of HBV exposure than in other Italian cohorts (Babudieri et al., 58%), where 87% of the examined population was male. Considering only the female population, the rate of anti-HBc positivity was much higher than in our cohort (46.0% vs. 19.9%) [24]. Compared to Pontali et al., the number of individuals with HBV exposure remains higher (77.4%) than in our data. It is essential to specify that the examined cohort represented only HIV-infected males, with a high proportion of PWID [57].

PWID and female sex workers are considered high-risk groups for BBI infections, including HIV, HCV, and HBV. PWIDs are usually infected through shared needles, syringes, and other infected injection equipment [58,59]. Incarcerated women used drugs more frequently, and harder ones, such as heroin, than men [28]. Female inmates have more difficulties than men in areas linked to substance abuse, such as educational background, childhood family–social environment, mental health, and physical health [28]. All these drug abuse-related socio-economic conditions could impact HBV exposure and adherence to BBI screening. Specifically, although women represent a minority among detainees [60], they have a high rate of HBV infections transmitted via intravenous drug use and sex [61]. Our low ratio of anti-HBc positive titer is probably related to the low proportion of PWID. Otherwise, PWID remains one of the most critical risk factors for HBV exposure, as indicated in multivariate analysis.

Analyzing the HBV vaccination immunological status, only 30% of patients had an anti-HBs protective titer, 38% had a non-protective anti-HBs titer, and 32% were not vaccinated. Rezende et al. showed a 40.8% prevalence of adequate vaccination, 48% of prisoners without a protective anti-HBs titer, and 39% without a history of immunization in the female population [35]. The data reported are like those evidenced in our study. Low coverage of protective anti-HBs titer in jail could represent a profound problem in health policy. The classic HBV vaccination schedule with three doses (at 0, 1, and 6 months) might not apply to all prisoners as they might be released anytime. As explained before, female prisoners, mainly when set free, could be at more risk of fragile conditions. Drug use and sexual abuse could be frequent experiences during this period. In prison, women can also be followed more closely for BBI screening and vaccination, but new immunization strategies should be implemented. Different accelerated vaccination schemes have been proposed for HBV vaccination, to facilitate vaccination adherence and ensure adequate coverage at the same time [62]. Gender can also affect vaccination response in adults. Trevisan et al. demonstrated that women had a greater anti-HBs titer than men after immunization, with a 1.21-fold increase in median antibody titer (*p* = 0.0023) [63].

The literature provides us with necessary information about the coverage of people with an anti-HBs titer below 10 mUI/mL during years following vaccination, highlighting the protective status of those individuals and the unnecessary need for revaccination, if not immunocompromised [64,65]. In our cohort of patients, 44 women were found with an anti-HBs titer below 10 mUI/mL, but data about the timeline of their HBs antibodies status were missing, so we cannot state whether they were protected or could be defined as “non-responders”, following the CDC criteria [66]. Patients with an HBs-titer < 10 mUI/mL exposed to a source of infection for HBV should receive a prompt post-exposure prophylaxis (PEP) with Hepatitis B immunoglobulin and vaccination, as indicated by the CDC [67]. The jail environment can represent the perfect situation to start the immunization with the scheduled doses, followed by anti-HBs titer testing to assess the need for a booster dose. In our study, we found that 37 women were not vaccinated. It could be a prospective aim to vaccinate this cohort and follow the HBs’ titer through time.

Our study shows an exciting point of view regarding women in jail; Italian data in this setting are scarce and, in many cases, the female cohort is under-represented. To our knowledge, this is one of the few HBV gender-based studies done in Italian correctional settings. Moreover, our research suggests that screening and HBV management policies in Italian jails can be crucial in preventing HBV transmissions and should be implemented.

Undoubtedly, there are some limitations. Firstly, there is the retrospective nature of the study. Missing data, such as a complete vaccination history or other socio-economic behaviors, could impact our analysis. In particular, sexual behavior could be investigated more appropriately, such as the presence of multiple partners, the frequency of sexual intercourse per year, and modalities of sex transmission. Additionally, incorporating family medical history and lifestyle factors, like tattooing, dental, or surgical procedures, is crucial as they are known transmission routes for HBV [68,69,70,71]. 

Another limitation of our study is the small sample size. The analysis presented interesting statistical results, but these could be strengthened by amplifying the number of patients involved. Nevertheless, our investigation can be the basis for future studies. Finally, it does not compare data between detained females and males, missing an opportunity to explore gender differences in HBV exposure and immunization.

As reported earlier, the future purpose could be a prospective cohort analysis with new strategies after implementing the HBV vaccination program.

Although active HBV infections are infrequent in our cohort, it could be interesting to analyze the HBV genotype. A high proportion of some genotypes, which are related to vaccine escape, could increase the risk of HBV transmission, even in the vaccinated and under-protected population [72,73,74,75,76]. Baseline screening implementation with other sexually transmitted diseases that could impact HBV transmission should be checked at the time of incarceration. In our study, we only tested HCV, HIV, and syphilis. Still, as in the case of other sexually transmitted diseases (STDs), such as herpes simplex virus 2 (HSV-2), chlamydia, gonorrhea, and other unspecified STDs, there is a correlation between those kinds of infections and HBV coinfection [77,78] which may be investigated deeper.

## 5. Conclusions

Further investigation is critical to better understand HBV infection dynamics among women in correctional facilities, a demographic that has been underrepresented in research. This study points to the necessity of examining disparities in HBV exposure and vaccination between genders in these environments. Aligning with the WHO’s hepatitis B eradication goals by 2030, our findings advocate for the urgent implementation of comprehensive HBV screening and vaccination programs in correctional settings, addressing this high-risk group more effectively.

## Figures and Tables

**Table 1:** General characteristics of 156 incarcerated women divided by anti-HBc status.

Variable	Total (156)	Anti-HBc − (125)	Anti-HBc + (31)	*p*
Age (years), Median (IQR)	41.0 (34.0–48.0)	41.0 (33.0–48.0)	43.0 (36.0–51.0)	0.06
Geographical area of origin, n (%)				0.02
Italy	91 (58.3)	80/91 (87.9)	11/91 (12.1)	
East-Europe	38 (24.4)	24/38 (63.2)	14/38 (36.8)	
Africa	16 (10.3)	12/16 (75.0)	4/16 (25.0)	
South-Central America	9 (5.8)	8/9 (88.9)	1/9 (11.1)	
Asia	2 (1.3)	1/2 (50.0)	1/2 (50.0)	
Anti-HCV positive, n (%)	33/156 (21.1)	25/33 (75.8)	8/33 (24.2)	0.47
HCV RNA positive	16/33 (48.5)	13/25 (52.0)	3/8 (37.5)	0.37
Anti-HIV positive, n (%)	4 (2.6)	4/4 (100.0)	0/4 (0.0)	0.22
HBV serological profile, n (%)				
HBsAg positive	2 (1.3)	0 (0.0)	2 (1.3)	0.04
Anti-HBs positive	118 (75.6)	88 (56.4)	30 (19.2)	0.02
Syphilis serological profile, n (%)				
VDRL	0 (0.0)	0 (0.0)	0 (0.0)	
TPHA	4 (2.9)	3 (1.9)	1 (0.7)	1.00
Transmission, n (%)				0.03
Sexual transmission	122 (78.2)	103 (66.0)	19 (12.2)	
PWID	28 (18.0)	19 (12.2)	9 (5.8)	
Unknown	5 (3.2)	3 (1.9)	2 (1.3)	
Transfusions	1 (0.6)	0 (0.0)	1 (0.6)	
Drug abuse/Substitutive therapy and other psychiatric treatments, n (%)				
Benzodiazepine	20 (12.8)	15 (9.6)	5 (3.2)	0.55
Methadone	10 (4.5)	7 (5.0)	3 (1.9)	0.42
Cocaine	6 (3.9)	6 (3.9)	0 (0.0)	0.60
Opium	4 (2.6)	4 (2.6)	0 (0.0)	0.59
Cannabis	2 (1.3)	2 (1.3)	0 (0.0)	1.00

IQR: interquartile range. PWID = people who inject drugs.

**Table 2:** Logistic regression analysis to assess the relationship between demographics, clinical characteristics and anti-HBc positivity.

Variable	Univariate Analysis		Multivariate Analysis	
	OR (95%IC)	*p*	OR (95%IC)	*p*
Age	1.03 (1.00–1.07)	0.05	1.06 (1.01–1.11)	0.011
Geographical area of origin				
Italy	Ref	Ref	Ref	Ref
North-East-Europe	4.24 (1.97–10.60)	0.002	11.67 (3.29–41.30)	<0.001
Africa	2.42 (0.66–8.85)	0.180	6.92 (1.51–31.60)	0.013
South-Central America	0.91 (0.10–7.98)		1.87 (0.19–18.70)	0.592
Asia	7.27 (0.42–124.80)		14.9 (0.80–278.00)	0.070
Anti-HCV positive	1.39 (0.56–3.48)	0.71		
Transmission				
Sexual intercourses	Ref	Ref	Ref	Ref
PWID	2.57 (1.01–6.52)	0.047	6.55 (1.96–21.9)	0.002
Transfusions	-	-	-	-
Unknown	3.61 (0.56–23.1)	0.175	4.00 (0.37–18.70)	0.251
Substitutive therapy or other psychiatric treatments				
Methadone	1.81 (0.44–7.42)	0.82		
Benzodiazepine	1.41 (0.47–4.23)	0.61		

**Table 3:** General characteristics of 115 incarcerated women divided by HBV seroprotection.

Variable	Total (115)	Anti-HBs < 10 mUI/mL (81)	Anti-HBs ≥ 10 mUI/mL (34)	*p*
Age (years), median (IQR)	39.0 (32.0–48.9)	41.0 (32.0–48.0)	37.0 (31.0–46.0)	0.37
Geographical area of origin, n (%)				0.04
Italy	71 (61.7)	47/71 (66.2)	24/71 (33.8)	
North-East-Europe	24 (20.9)	21/24 (87.5)	3/24 (12.5)	
Africa	12 (10.4)	10/12 (83.3)	2/12 (16.7)	
South-Central America	7 (6.1)	3/7 (42.9)	4/7 (57.1)	
Asia	1 (0.9)	0/1 (0.0)	1/1 (100)	
Anti-HCV +, n (%)	20 (17.4)	13/20 (65.0)	7/20 (35.0)	0.60
HCV RNA positive (%)	7/20 (35.0)	3/13 (53.8)	4/7 (57.1)	0.09
Anti-HIV +, n (%)	2 (1.7)	1/2 (50.0)	1/2 (50.0)	0.51
Syphilis serological profile, n (%)				
VDRL	0 (0.0)	0 (0.0)	0 (0.0)	
TPHA	3 (2.6)	2 (1.7)	1 (0.9)	1.00
Trasmission, n (%)				0.26
Sex	98 (85.2)	71 (61.7)	27 (23.5)	
PWID	17 (14.8)	10 (8.7)	7 (6.1)	
Drug abuse/Substitutive therapy and other psychiatric treatments, n (%)				
Benzodiazepine	14 (12.1)	10 (8.7)	4 (3.4)	1.00
Methadone	6 (5.2)	3 (2.6)	3 (2.6)	0.36
Cocaine	6 (5.2)	2 (1.7)	4 (3.5)	0.04
Oppium	4 (3.5)	2 (1.7)	2 (1.7)	0.36
Cannabis	2 (1.7)	1 (0.9)	1 (0.9)	0.52

IQR: Interquartile Range; PWID = people who inject drugs.

**Table 4:** Logistic regression analysis to assess the relationship between demographics and clinical characteristics for the absence of HBV seroprotection.

Variable	Univariate Analysis		Multivariate Analysis	
	OR (95%IC)	*p*	OR (95%IC)	*p*
Age (years)	1.02 (0.98–1.05)	0.32	1.03 (0.99–1.08)	0.103
Geographical area of origin				
Italy	Ref.	Ref.	Ref.	Ref.
North-East-Europe	3.57 0.97–13.2)	0.056	4.55(1.19–17.50)	0.027
Africa	2.55 (0.52–12.6)	0.250	3.08 (0.61–15.60)	0.174
South-Central American	0.38 (0.08–1.85)	0.233	0.44 (0.08–2.720)	0.103
Asia	-	-	-	-
Anti-HCV positive	0.73 (0.27–2.05)	0.56		
Anti-HIV positive	0.41 (0.03–6.79)	0.54		
History of endovenous drug injections	0.54 (0.19–1.57)	0.26		
Substitutive therapy				
Methadone	0.40 (0.08–2.07)	0.27		

## Data Availability

Data will be available upon specific request.
